# Supplementary material for: Ivermectin Inhibits HBV Entry into the Nucleus by Suppressing KPNA2
Source: Viruses. 2022 Nov 8;14(11):2468. doi: 10.3390/v14112468 (PMC9695645; doi:10.3390/v14112468)
Supplement: Supplementary file 1 [file viruses-14-02468-s001.zip › viruses-1954996-supplementary.pdf]

Supplementary Materials and Methods

PreS1 binding and internalization assay

The protocol was modified from that of Iwamoto et al. [64]. HepG2-hNTCP-C4 cells were inoculated with 40 nM preS1-TAMRA at 4°C for 1 h to allow attachment of the peptide to the cell surface. Then, the cells were transferred to 37°C for 8 h to allow the incorporation of preS1-TAMRA into the cells. PreS1 internalization was observed using a BZ-X810 all-in-one fluorescence microscope (Keyence, Osaka, Japan).

References

64. Iwamoto, M.; Saso, W.; Sugiyama, R.; Ishii, K.; Ohki, M.; Nagamori, S.; Suzuki, R.; Aizaki, H.; Ryo, A.; Yun, J.H.; et al. Epi-dermal growth factor receptor is a host-entry cofactor triggering hepatitis B virus internalization. *Proc. Natl. Acad. Sci. USA.* **2019,** *116*, 8487–8492.

Supplementary Data

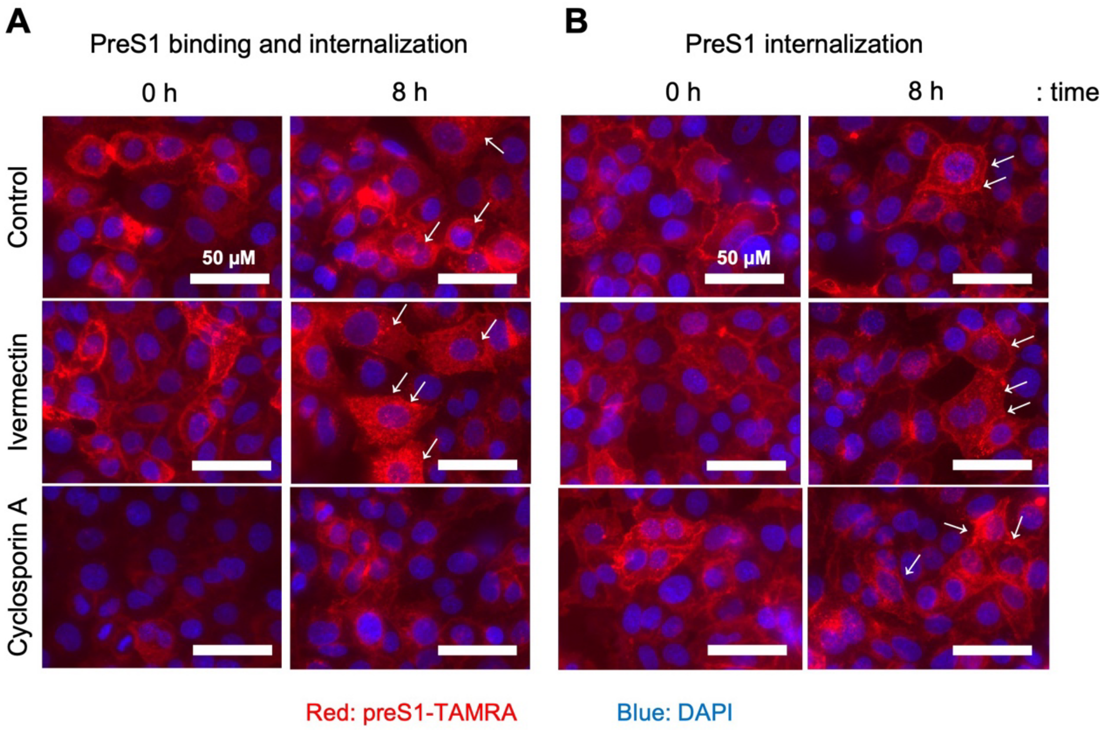

Figure S1. Ivermectin does not inhibit preS1 binding or uptake. HepG2-hNTCP-C4 cells were inoculated with 40 nM preS1-TAMRA at 4°C for 1 h and then reacted at 37°C for 8 h. (**A**) Fluorescence imaging of preS1-TAMRA in the preS1 binding and internalization assay. The cells were treated with 10 μM ivermectin, 10 μM cyclosporin A, or 0.1% DMSO (vehicle control) from the 4°C step. (**B**) Fluorescence imaging of preS1-TAMRA in the preS1 internalization assay. The cells were treated with 10 μM ivermectin, 10 μM cyclosporin A, or 0.1% DMSO (vehicle control) from the 37°C step. Arrows indicate the cells in which preS1-TAMRA was internalized.

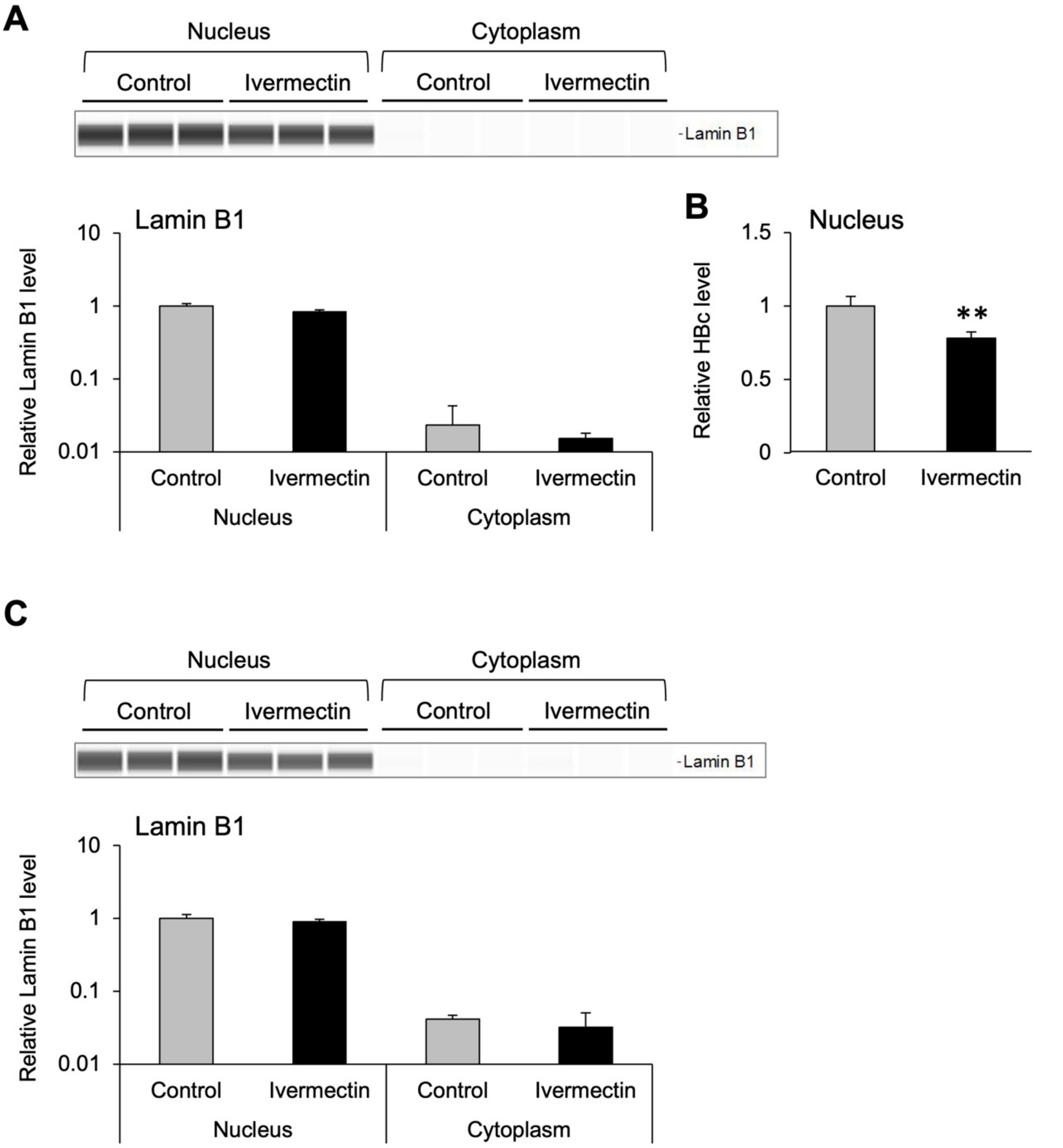

Figure S2. Confirmation of nuclear and cytoplasmic separation. (**A**) Quantification of Lamin B1 (a nuclear marker) expression in the nucleus and cytoplasm of HepG2-hNTCP-C4 cells after 48 h of HBc transfection. HepG2-hNTCP-C4 cells were treated with 5 μM ivermectin or 0.1% DMSO (vehicle control) from 24 h before to 48 h after HBc transfection. Expression was corrected for the total protein levels of each sample. (**B**) Quantification of HBc expression in the nucleus of HepG2-hNTCP-C4 cells after 48 h of HBc transfection. HBc expression in Fig. 4B was corrected for Lamin B1 expression in Supplemental Fig. 2A. (**C**) Quantification of nuclear and cytoplasmic Lamin B1 expression after treating HepG2-hNTCP-C4 cells with 10 μM ivermectin for 48 h. Expression was corrected for the total protein levels of each sample. In the graphs, the protein expression of the control was defined as 1. Data are presented as the mean ± SD ($n = 3$). $^{**}p < 0.01$.

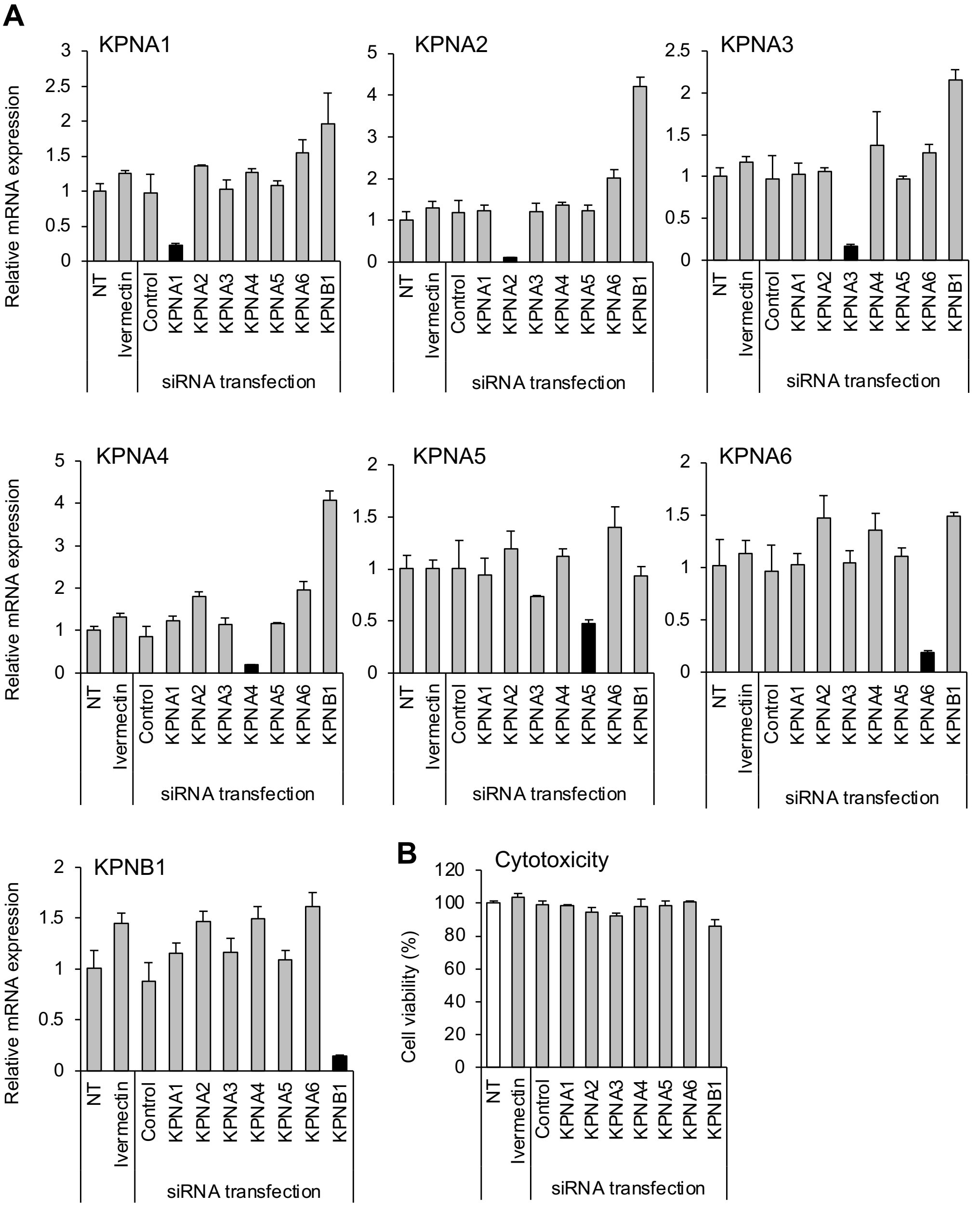

Figure S3. Gene expression of each importin and cell viability after siRNA transfection. HepG2-hNTCP-C4 cells were treated with 10 μM ivermectin or transfected with siRNA. (A) Depletion efficiency of each importin 5 days after siRNA transfection. Importin *α* (karyopherin *α*; KPNA) family and importin β (karyopherin β1; KPNB1) siRNAs (each 20 nM) were transfected at the time of cell seeding. NT, non-transfection. The mRNA expression in the NT group was defined as 1. (B) Cell viability after 9 days of HBV infection (12 days after siRNA transfection). The absorbance in the NT group was defined as 100%. Data are presented as the mean ± SD (*n* = 3).

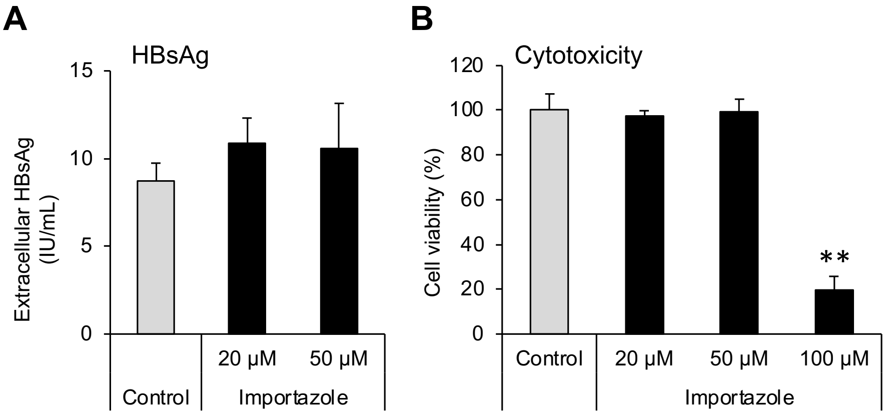

Figure S4. Effect of high concentrations of importazole on HBV infection. (A) Extracellular HBsAg levels 9 days after HBV infection. HepG2-hNTCP-C4 cells were treated with different concentrations of importazole or 3% DMSO (control) for 48 h. (B) Cell viability 9 days after HBV infection. The absorbance of the control group was defined as 100%. Data are presented as the mean ± SD (*n* = 3).
